# Responsibility of regulatory gene expression and repressed protein synthesis for triacylglycerol accumulation on sulfur-starvation in *Chlamydomonas reinhardtii*

**DOI:** 10.3389/fpls.2014.00444

**Published:** 2014-09-11

**Authors:** Atsushi Sato, Rie Matsumura, Naomi Hoshino, Mikio Tsuzuki, Norihiro Sato

**Affiliations:** ^1^School of Life Sciences, Tokyo University of Pharmacy and Life SciencesHachioji, Japan; ^2^Japan Science and Technology Agency, Core Research for Evolutionary Science and TechnologyChiyoda-ku, Japan

**Keywords:** acyltransferases, *Chlamydomonas reinhardtii*, lipid droplets, *SAC1*, sulfur-starvation, *SNRK2.2*, protein synthesis, triacylglycerol

## Abstract

Triacylglycerol (TG) synthesis is induced for energy and carbon storage in algal cells under nitrogen(N)-starved conditions, and helps prevent reactive oxygen species (ROS) production through fatty acid synthesis that consumes excessive reducing power. Here, the regulatory mechanism for the TG content in sulfur(S)-starved cells of *Chlamydomonas reinhardtii* was examined, in comparison to that in N- or phosphorus(P)-starved cells. S- and N- starved cells exhibited markedly increased TG contents with up-regulation of mRNA levels of diacylglycerol acyltransferase (DGAT) genes. S-Starvation also induced expression of the genes for phosphatidate synthesis. In contrast, P-starved cells exhibited little alteration of the TG content with almost no induction of these genes. The results implied deficient nutrient-specific regulation of the TG content. An *arg9* disruptant defective in arginine synthesis, even without nutritional deficiencies, exhibited an increased TG content upon removal of supplemented arginine, which repressed protein synthesis. Repression of protein synthesis thus seemed crucial for TG accumulation in S- or N- starved cells. Meanwhile, the results of inhibitor experiments involving cells inferred that TG accumulation during S-starvation is supported by photosynthesis and de novo fatty acid synthesis. During S-starvation, *sac1* and *snrk2.2* disruptants, which are defective in the response to the ambient S-status, accumulated TG at lower and higher levels, respectively, than the wild type. The *sac1* and *snrk2.2* disruptants showed no or much greater up-regulation of DGAT genes, respectively. In conclusion, TG synthesis would be activated in S-starved cells, through the diversion of metabolic carbon-flow from protein to TG synthesis, and simultaneously through up-regulation of the expression of a particular set of genes for TG synthesis at proper levels through the actions of *SAC1* and *SNRK2.2*.

## Introduction

Triacylglycerol (TG), which is one of the neutral lipids, is ubiquitous in eukaryotes and also present in a limited group of prokaryotes (Athenstaedt and Daum, 2006). Distinct from polar lipids that are predominantly membrane components, TG is localized in a specific organelle designated as lipid droplets. TG has been considered as a storage compound: e.g., in oil seed plants, fatty acids esterified to TG in seeds undergo β-oxidation for energy production and gluconeogenesis, which supports the seedling growth after germination (Quettier and Eastmond, 2009). However, recent studies indicated that TG also participates in the synthesis of membrane lipids as an intermediate metabolite by supplying fatty acids in actively growing cells of yeast, *Saccharomyces cerevisiae*, and thus is critical for maintenance of lipid homeostasis (Rajakumari et al., 2010; Kohlwein and Henry, 2011). On the other hand, from industrial aspects, TG is important as a food oil and has been recently expected to be a source of biodiesel fuel (BDF), which is produced through its chemical conversion into methyl or ethyl esters of fatty acids. Extensive attention has been paid to BDF production with photosynthetic organisms in particular, in terms of the carbon neutrality concept (Durrett et al., 2008; Hu et al., 2008).

Many algal species have several advantages over terrestrial plants as to the production of biomaterials, including high annual biomass productivity on an area basis that is ensured by their high growth rates (Hu et al., 2008). Eukaryotic algae in general contain TG at a low level during optimal growth conditions, but the content remarkably increases with culture aging (Dunstan et al., 1993) or under ambient stress conditions such as nutritional limitation, high salinity (Siaut et al., 2011), or high light (Khotimchenko and Yakovleva, 2004). It has been consistently shown that, as to nutrients, nitrogen deficiency is markedly effective for induction of accumulation of TG in numerous algal species including green and red algae, diatoms, golden algae, haptophytes, eustigmatophytes, dinophytes, and yellow-green algae (see a review by Hu et al., 2008). Meanwhile, the effects of deficiencies of other nutrients on the TG content were investigated, especially in a green alga, *Chlamydomonas reinhardtii*, distinct results concerning sulfur-starved cells being reported in the literature: Boyle et al. (2012) reported marked induction of TG accumulation whereas Fan et al. (2012) observed little induction. Phosphorus (P)-deficiency seemed to have no or only a minor effect, if any, on the increase in the TG content (Boyle et al., 2012; Fan et al., 2012).

Study of the mechanism by which the accumulation of TG is stimulated, e.g., identification of key genes as to the synthesis of TG and proteomic analysis of lipid droplets, is indispensable for enhancement of the productivity of TG, but this has only just begun for algal species (Nguyen et al., 2011; Boyle et al., 2012; Deng et al., 2012; Msanne et al., 2012). The genes responsible for the terminal step of the synthesis of TG include those for diacylglycerol acyltransferases (DGATs), which could be important players determining the accumulated level of TG. The genome of *C. reinhardtii* contains one homolog of *DGAT1* and five homologs of *DGAT2* designated as *DGTT1-5* (Merchant et al., 2012). Expression of *DGAT1* and *DGTT1-4* was induced at the transcript level in response to N- or S-starvation, but was little affected by P-starvation (Miller et al., 2010; Boyle et al., 2012; Blaby et al., 2013; Ramanan et al., 2013).

Sulfoquinovosyl diacylglycerol (SQDG) is one of the membrane lipids specific to chloroplasts, and is responsible for the structural and functional integrity of the photosystem II complex in *C. reinhardtii* (Sato et al., 1995, 2003a; Minoda et al., 2003; Sato, 2004). Intriguingly, we recently reported that cells of *C. reinhardtii* degrade SQDG for utilization of it as a major intracellular sulfur (S)-source for the synthesis of protein, especially at an early phase of S-starvation (Sugimoto et al., 2007, 2008, 2010). In the course of that study, we noticed that S-deficiency stress as well as N-limiting stress induces pronounced accumulation of TG. *C. reinhardtii* is suitable for gene manipulation, and abundant mutants defective in a variety of physiological processes are available (Rochaix, 1995). Moreover, its whole genome has been successfully sequenced (Merchant et al., 2007). *C. reinhardtii* would thus be a strong biological tool for elucidation of the mechanism by which the accumulated level of TG is enhanced.

S is one of the macronutrients, and is incorporated into plants mainly as sulfate. A greater part of study on plant behavior in response to the ambient S-status has been restricted to green plants such as a seed plant, *Arabidopsis thaliana*, and a green alga, *C. reinhardtii*. These two organisms when exposed to S-limitation exhibit similar up-regulation of expression of the genes for acquisition of external S such as that of the sulfate transporter, and also for primary S-assimilation including the synthesis of cysteine (Nikiforova et al., 2003; Zhang et al., 2004; Toepel et al., 2011; Hubberten et al., 2012). In *C. reinhardtii*, in particular, regulatory components including the SAC1 (Sulfur Acclimation 1; Davies et al., 1996) and SNRK2.2 (SNF1-related protein kinase 2.2, previously known as SAC3; Davies et al., 1999; Moseley et al., 2009) proteins have been identified as components of the signaling pathway responsible for the cellular response to the ambient S-status. The SAC1 protein is homologous to anion transporters of other organisms such as the mammal Na^+^/SO^2−^_4_ transporter whereas SAC1 like transporters designated as SLT1 and 2 seem to function as high-affinity sulfate transporters at the plasma membrane in *C. reinhardtii* (Davies et al., 1996; Pootakham et al., 2010). SAC1 is thus postulated to sense a shortage of an external S-source, and then to transduce the signal for up-regulation of the transcript levels of a special set of genes (Davies et al., 1996; Zhang et al., 2004; Moseley et al., 2009). On the other hand, the SNRK2.2 protein belongs to the serine/threonine kinase group, and seems to either positively or negatively regulate physiological responses related to the ambient S-status (Davies et al., 1999; Zhang et al., 2004; Moseley et al., 2009). The degradation of SQDG induced by S-starvation was performed by the use of both the SAC1 and SAC3 proteins as positive regulators (Sugimoto et al., 2010). It is thus of interest whether or not, or how these factors are involved in the above-mentioned TG accumulation under S-starved conditions.

Here, we investigated alterations in the TG content and the expression levels of the genes for the synthesis of TG in cells of *C. reinhardtii* during S-starvation, and compared them with those during N- or P-starvation, to reveal the deficient-nutrient dependent regulatory mechanism for the level of TG accumulation. The mechanism by which the accumulation of TG is stimulated under S-starved conditions will be discussed in view of the actions of the *SAC1* and *SNRK2.2* genes, and repression of global protein synthesis.

## Materials and methods

### Strains and growth conditions

The *C. reinhardtii* strains used were CC-125 as the wild type, and three disruptants as to the *SAC1* (CC-3794), *SNRK2.2* (CC-3799), and *ARG9* (CC-4440) genes, and the respective complemented strains (CC-3795, CC-3798, CC-4441). These disruptants and complemented strains were purchased from the *Chlamydomonas* Resource Center (http://chlamycollection.org/). *Chlorella kessleri* 11 h was also used (Sato et al., 2003b). Cells were cultured with continuous illumination at 30°C in TAP medium (Gorman and Levine, 1965) for mixotrophic growth of *C. reinhartdii* cells in a flask on a rotary shaker, or in 3/10 HSM (Sueoka, 1960) for photoaoutotrophic growth of *C. reinhardtii* and *C. kessleri* cells in an oblong glass vessel with aeration. A disruptant as to the *ARG9* gene for N-acetyl ornithine aminotransferase in chloroplasts (*arg9*), which is deficient in the synthesis of arginine, was cultured with supplementation of arginine (0.57 mM; Remacle et al., 2009). For transfer to nutritionally starved conditions, cells grown to the mid-logarithmic phase in TAP medium or ones grown to the mid-linear phase in 3/10 HSM were harvested by centrifugation, washed twice and then resuspended in the corresponding S-, N-, or P-free medium. The S- and P-free media were prepared by replacing sulfate and phosphate, respectively, with chloride, whereas the N-free medium was prepared by substituting potassium for ammonium. Growth of the cells was monitored by determination of the optical density at 730 nm with a spectrophotometer, DU640 (Beckman, USA). When needed, the flask, in which cells of *C. reinhardtii* were mixotrophically cultured, was completely covered with aluminum foil to obtain dark conditions.

### Inhibitors of protein synthesis, fatty acid synthesis, and photosynthesis

An inhibitor, such as chloramphenicol (CAP, 100 μg ml^−1^), cycloheximide (CHI, 8 μg ml^−1^), cerulenin (10 μM), or 3-(3,4-dichlorophenyl)-1,1-dimethylurea (DCMU, 50 μM), was added at the respective final concentrations, immediately after the change from nutrient-repleted to –depleted medium. Methanol was used as a carrier of CAP (final concentration, 1%). CAP and CHI are inhibitors of de novo protein synthesis on chloroplast and mitochondrial 70S ribosomes, and cytoplasmic 80S ribosomes, respectively. Cerulenin is an inhibitor of de novo synthesis of fatty acids, whereas DCMU inhibits photosynthesis.

### Extraction of lipids, proteins, and chlorophyll (Chl) and their quantitative analysis

Total lipids were extracted from cells of *C. reinhardtii* or *C. kessleri*, according to the method of Bligh and Dyer (1959), and thereafter separated into individual neutral lipid classes by TLC on precoated silica gel plates (Merck 5721) with a solvent system of hexane/diethylether/acetate (70:30:1, v/v/v). The spots of lipids were visualized by illumination with UV light after spraying with primulin (0.01% in 80% acetone, w/v). Fatty acid methyl esters were prepared from the total lipids, TG, and free fatty acids (FFA) by heating at 95°C with 5% anhydrous methanolic HCl, and thereafter analyzed by capillary GLC, as described previously (Sato et al., 1995). The fatty acid content of each fraction was estimated with arachidonic acid as an internal standard. The TG content relative to total lipids was expressed as mol% on the basis of included fatty acids. On the other hand, whole-cell extracts were prepared through disruption of cells in an extraction buffer (50 mM Tris-HCl, pH 7.5, 0.5% Triton X-100) with a Beads Crusher mT-12 (TAITEC, Japan), and thereafter the protein contents were measured with a BCA assay kit (Pierce, USA). Chl was extracted from cells with methanol and its content was measurement by spectroscopy, according to the method of Grimme and Boardman (1972).

### Microscopic observation of lipid droplets and starch granules

A Nile red solution (0.25 mg·ml^−1^ in acetone) was added to cell suspensions of *C. reinhardtii* and *C. kessleri* (1:50, v/v), and the stained cells were observed under a fluorescence microscope (BX-FLA; Olympus Optical Co., Tokyo, Japan) with the use of a 520–550 nm excitation filter. Meanwhile, a solution of 0.1% KI containing 0.1% I_2_ was added to a cell suspension of *C. reinhardtii* (1:3, v/v) for staining of starch granules, which were thereafter observed microscopically, as described by Izumo et al. (2011).

### Semi-quantitative determination of transcript levels by reverse transcriptase (RT)-PCR

Total RNA was extracted and purified by phenol–chloroform extraction, as described by Los et al. (1997), and then used for the synthesis of cDNA by reverse-transcription with random primers (Tabei et al., 2007). The cDNA synthesized was used as a template for semi-quantitative RT-PCR (Tabei et al., 2007). The specific forward (F) and reverse (R) primers specific to the respective genes are shown in the supplementary material, with available ID numbers for the transcripts.

The primer sets for *DGAT1* and *DGTT1-4, GPDH1-3*, and 18 rRNA were the same as those reported by Msanne et al. (2012), Herrera-Valencia et al. (2012), and Teramoto et al. (2002), respectively. The primer sets for *GPAT1, GPAT2*, and *LPAAT* were designed on the basis of the corresponding cDNA sequences. The amplified DNA fragments were subjected to agarose gel electrophoresis, and a fluorescent image of a gel after staining with ethidium bromide was obtained by photography. The fluorescence intensities of DNA bands were quantified with ImageJ (http://rsbweb.nih.gov/ij/). As regards the respective genes, the values were estimated relative to that of 18S rRNA as an internal control.

## Results

### Enhanced accumulation of TG under s-starved conditions in *C. renhardtii* and *C. kessleri*

**Figure 1:**
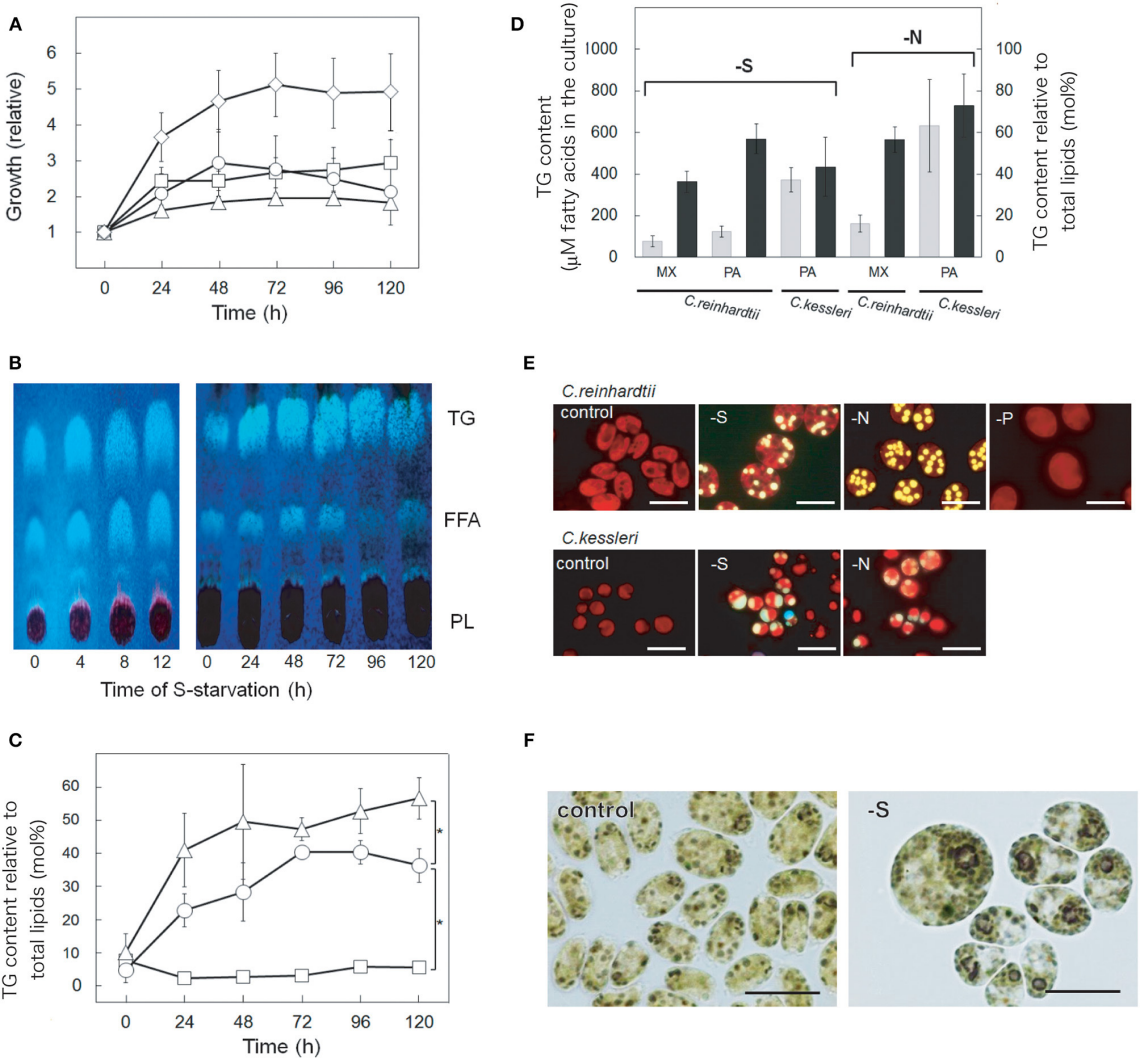
**Accumulation of TG in *C. reinhardtii* and *C. kessleri* cells. (A)** Cell growth of *C. reinhardtii* in TAP (open diamonds), TAP-S (open circles), TAP-N (open triangles), and TAP-P (open squares) medium. OD_730_ values relative to those at 0 h are shown. **(B)** A TLC profile of lipids in *C. reinhardtii* cells during S-starvation for a short term (up to 12 h, left panel) or long term (up to 120 h, right panel). Total lipids isolated from cells in 30-mL culture were loaded. FFA, free fatty acids. PL, polar lipids. **(C)** Changing patterns of TG contents relative to total lipids on the basis of fatty acids in cells of *C. reinhardtii* starved of S, N, or P. The symbols are the same as in **(A)**. **(D)** Accumulation of TG caused by S- or N-starvation for 120 h in *C. reinhardtii* and *C. kessleri*. The TG content of cells was expressed as that in the cultures (gray bars), and that relative to total lipids (black bars), respectively, on the basis of fatty acids. MX and PA indicate mixotrophic and photoautotrophic culturing conditions, respectively. **(E)** Marked accumulation of Nile-red stained lipid droplets that emitted yellow fluorescence in *C. reinhardtii* and *C. kessleri* cells starved of S or N for 120 h. **(F)** Induction of I_2_-stained starch accumulation in *C. reinhardtii* cells by S-starvation for 24 h. The values shown in **(A–D)** are the averages ± SE for three distinct groups of data. The significance of differences was evaluated by means of Student's *t*-test. ^*^P <0.05.

**Figure 2:**
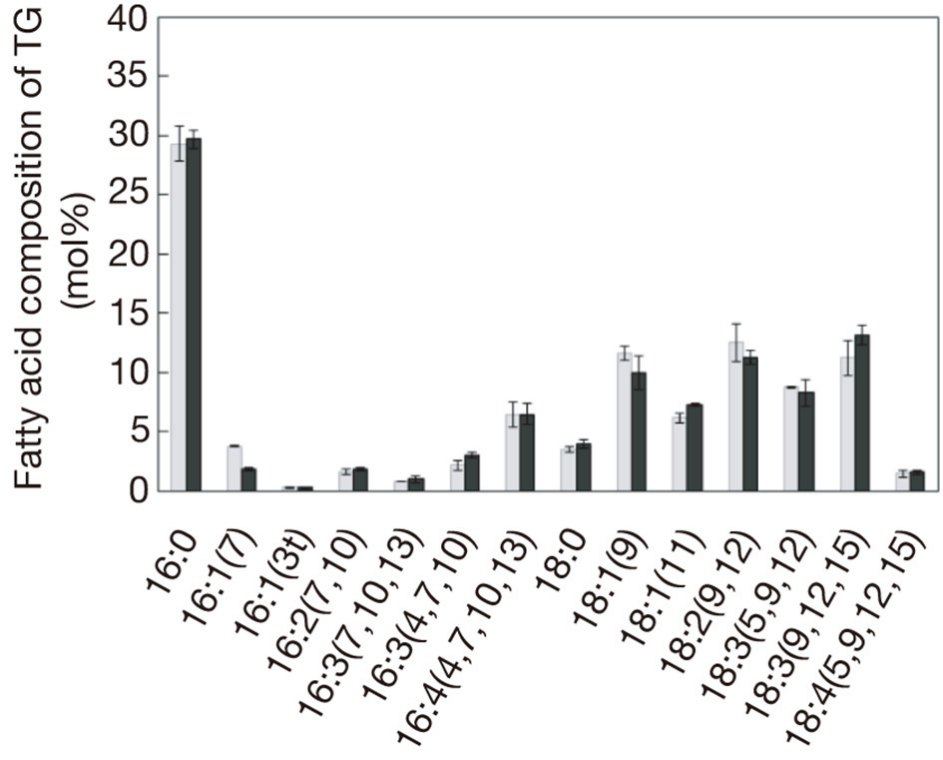
**The composition of constituent fatty acids of TG isolated from cells starved of S (gray bars) or N (black bars)**. The values are the averages ± SE for three distinct groups of data.

**Figure 3:**
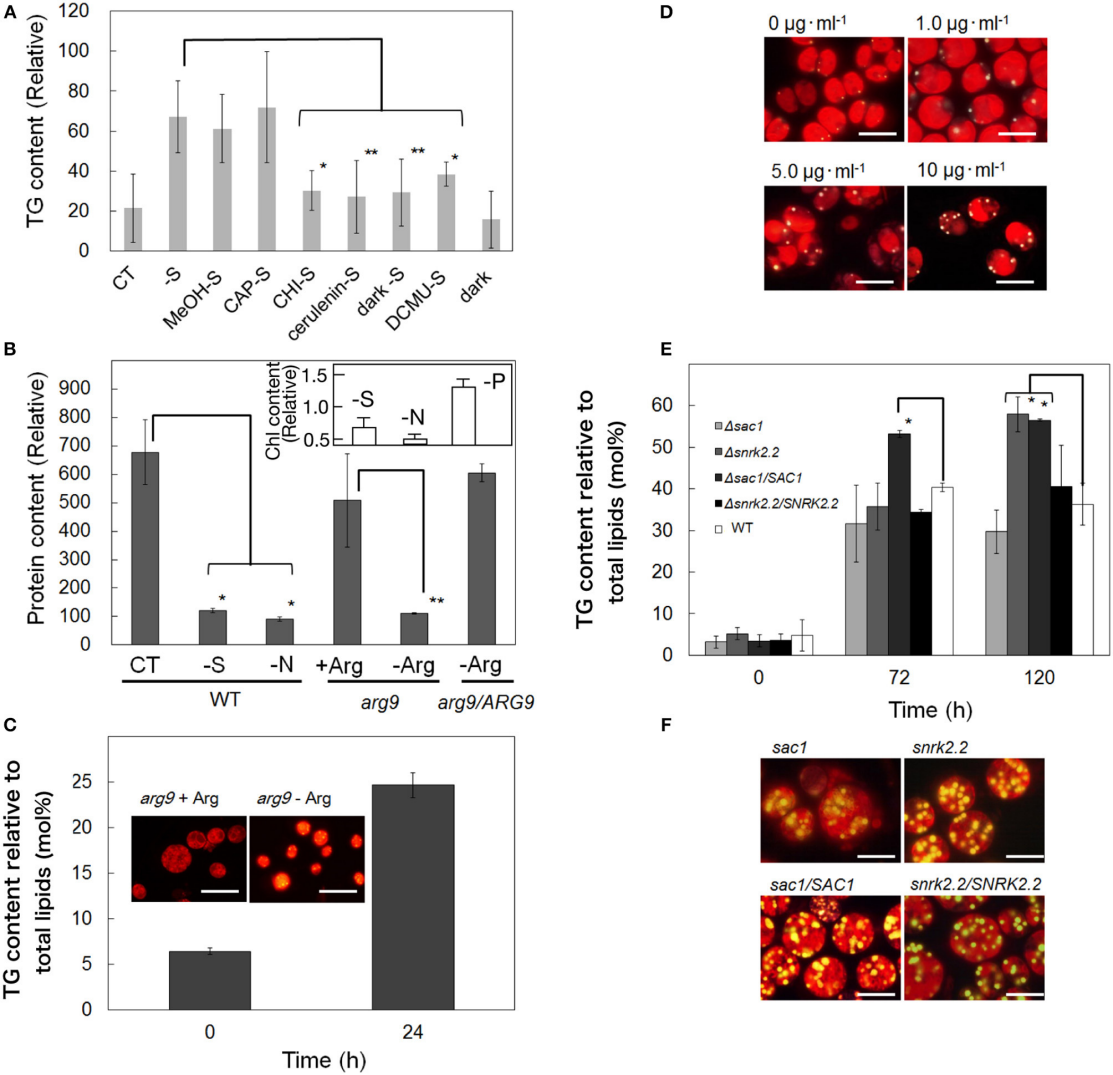
**Effects of inhibitors, light conditions, and mutations on the cellular content of TG in *C. reinhardtii*. (A)** TG contents in cells that were treated with inhibitors or placed under dark conditions, and then starved of S for 24 h. CT, control. MeOH, methanol used as a carrier of CAP. The values were estimated as fatty acid contents of TG per OD_730_ values. **(B)** Contents of total cellular proteins in cells of WT, an *arg9* disruptant, and a complemented strain as to *arg9* (*arg9*/*ARG9*) after 24-h culturing under normal or stress conditions. The values were estimated relative to those at 0 h. CT, –S, and -N indicate normal, and S- and N-starved conditions for the WT cells, respectively. +Arg and –Arg represent supplementation and non-supplementation of arginine, respectively, for the cultures of *arg9* and *arg9*/*ARG9* cells. Inset, the Chl content in WT cells starved for S, N, or P for 24 h. The values were estimated relative to those at 0 h. **(C)** An increase in the content of TG or lipid droplets in the *arg9* disruptant after 24-h culture in arginine-free medium. The values were estimated as TG content relative to total lipids on the basis of fatty acids. **(D)** Marked accumulation of Nile-red stained lipid droplets in cells upon treatment with CHI (1–10 μg·mL^−1^) for 24 h. S-Starvation induced accumulation of TG **(E)** or Nile-red stained lipid droplets **(F)** in mixotrophically grown cells of the *sac1* and *snrk2.2* disruptants, and complemented strains of *sac1*/*SAC1* and *snrk2.2*/*SNRK2.2*. The values **(E)** were estimated as TG content relative to total lipids on the basis of fatty acids. The values for the wild type **(E)** were the same as those in Figure 1C. The values shown in **(A–C,E)** are the averages ± SE for three distinct groups of data. The significance of differences was evaluated by means of Student's *t*-test. ^*^P < 0.05. ^**^P < 0.1.

**Figure 4:**
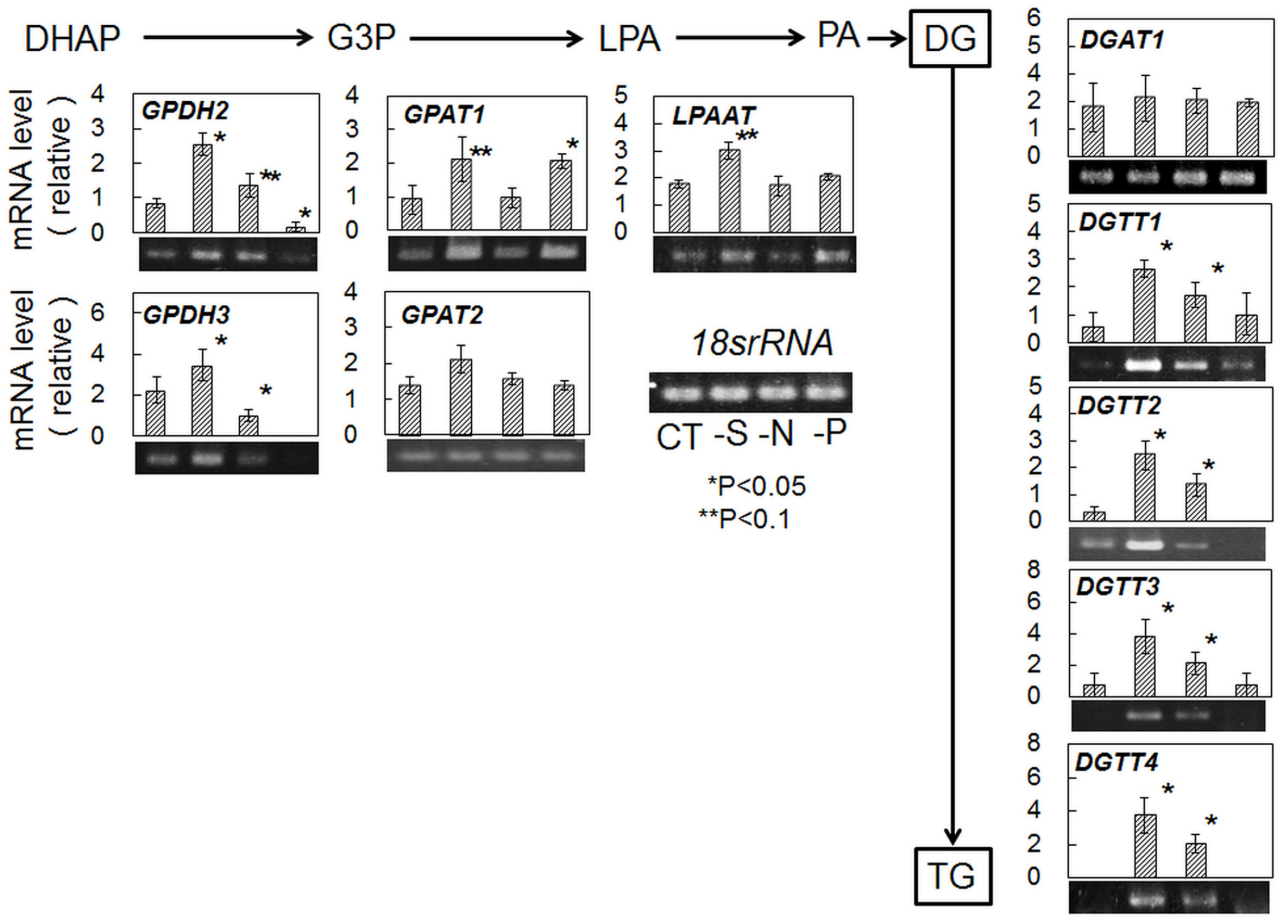
**Semi-quantitative RT-PCR analysis of mRNA levels of the genes for TG synthesis in *C. reinhardtii* cells starved of S, N or P for 8 h**. CT indicates normal conditions. The values were estimated from the intensities of the DNA bands corresponding to mRNAs of the respective genes for TG synthesis relative to that of 18s rRNA, as described under Materials and Methods. DHAP, dihydroxyacetone phosphate; G3P, glycerol 3-phosphate; LPA, lysophosphatidate; PA, phosphatidate; GPDH, G3P dehydrogenase; GPAT, G3P acyltransferase; LPAAT, lysophosphatidate acyltransferase; DGAT, diacylglycerol acyltransferase type I; DGTT, diacylglycerol acyltransferase type II. The values are the averages ± SE for three distinct groups of data. The significance of differences was evaluated by means of Student's *t*-test. ^*^P < 0.05. ^**^P < 0.1.

We first examined whether or not S-deficiency stress has an impact on the contents of non-polar lipids. The mixotrophic growth of cells of *C. reinhardtii* was retarded upon exposure to S-starvation, which finally resulted in an only 2.1-fold increase at 120 h (cf. a 4.9-fold increase under normal conditions, Figure 1A). TLC analysis of total lipids from *C. reinhardtii* cells showed the separation of non-polar lipid classes, TG and FFA, on a plate, from polar lipid classes that migrated much more slowly as a group with chlorophyll (Figure 1B). TG and FFA accounted for merely 5–10 mol% (see 0 h in Figure 1C) and 2–4 mol% (data not shown), respectively, relative to total lipids on the basis of fatty acids, under normal growth conditions. However, after the onset of S-starvation, the TG content began to increase as early as at 4 h, and thereafter accounted maximally for 40.3 mol% at 72 h, relative to total lipids, on the basis of fatty acids (Figures 1B,C). Accordingly, the TG content in the culture finally reached a level corresponding to 77 μM fatty acids (Figure 1D). In contrast, S-starvation had only a minor effect on the content of FFA (Figure 1B). Consistent with the enhanced accumulated level of TG, the S-starved cells eventually contained a substantial amount of lipid droplets, which appeared as intracellular globules that emitted yellow fluorescence against the background of red autofluorescence of Chl (Figure 1E, see –S for *C. reinhardtii*). Simultaneously, we observed that S-starved cells, but not S-repleted ones, markedly accumulated starch granules on staining of them with I_2_ (Figure 1F). S-Starvation under photoautotrophic growth conditions also caused drastic accumulation of TG to a level corresponding to 123 μM fatty acids in the culture or 56.9 mol% relative to total lipids on the basis of fatty acids (Figure 1D).

The effects of depletion of other macronutrients such as N and phosphorus (P) on the TG content were then examined. N-Starvation, as compared with S-starvation, retarded mixotrophic cell growth more severely (Figure 1A), with more pronounced elevation of the TG content to 56.6 mol% relative to total lipids, on the basis of fatty acids, or 162 μM fatty acids in the culture (Figures 1C,D). P-Starved mixotrophic cells showed similar delayed growth to S-starved ones, at least for 96 h (Figure 1A), however, the TG content increased little (Figure 1C). Compatible with these observations, the content of lipids droplets was greatly increased in N-starved cells, but was maintained at a low level in P-starved ones (Figure 1E). Overall, it was concluded that S-starvation is a strong induction factor for the accumulation of TG in *C. reinhardtii*, irrespective of whether the cells are mixotrophically or photoautotrophically grown, although the effect is less outstanding than that of N-starvation. Similarly, S-starvation caused *C. kessleri* cells to accumulate abundant TG with the appearance of lipid droplets (Figures 1D,E, see –S for *C. kessleri*), however, the TG accumulation was lower than with N-starvation (Figure 1D, compare –S with –N for *C. kessleri*).

In *C. reinhardtii*, the constituent fatty acids of TG that accumulated under S-starved conditions were composed of C16 and C18 acids, which respectively accounted for ca. 44 mol% and 56 mol% (Figure 2). C16 acids consisted of 16:0 (29 mol%) and mono- to tetra-unsaturated acids (totally, 15.2 mol%), whereas C18 acids were composed of 18:0 (3.6 mol%) and mono- to tetra-unsaturated acids (totally, 52.0 mol%). The fatty acid profile of TG in N-starved cells was found to be almost identical to that in S-starved cells.

### Effects of inhibitors of some physiological processes, light conditions, and respective mutations that cause a deficiency in arginine synthesis and an aberrant cellular response to the ambient S-status on the accumulated level of TG in cells of *C. reinhardtii*

We then characterized the induction system for the accumulation of TG under S-starved conditions in cells of *C. reinhardtii*, with the use of the inhibitors of protein synthesis, fatty acid synthesis, and photosynthesis, and light conditions (Figure 3A). Application of CHI resulted in almost complete repression of the accumulation of TG, whereas CAP had no effect. It thus was likely that the enhanced accumulation of TG under S-starved conditions depends on de novo synthesis of protein(s) encoded on the nuclear genome, and not on the chloroplast or mitochondria genome. On the other hand, as cerulenin prevented the elevation in the TG content, the elevation was implied to require de novo synthesis of fatty acids. Dark conditions and DCMU, respectively, repressed the increase in the TG content, inferring that light as an energy source for photosynthesis is indispensable for the enhanced accumulation of TG.

The Chl content, which reflects the level of the PSI and PSII complexes, was decreased at 24 h to 0.7- and 0.5-fold of the initial level in S- and N-starved cells, respectively (Figure 3B, inset). In contrast, the Chl content was increased in P-starved cells, although the increased level (1.3-fold of the initial level, Figure 3B, inset) was lower than in normal cells (a 3.6-fold increase, data not shown). Simultaneously observed was severe repression in accumulation of total cellular proteins in S- or N-starved cells (Figure 3B, see –S or –N, cf. Control). These results indicated that global protein synthesis was so severely repressed in S- and N-starved cells such that degradation of the PS complexes became necessary, but not in P-starved cells, which could still continue to construct the PS complexes. The stimulating effects of S- and N-starvation, but not of P-starvation, on the accumulation of TG might explain that the severe repression of global protein synthesis is a prerequisite for the stimulation of TG synthesis. To examine this possibility, first, we utilized the *arg9* disruptant of *C. reinhardtii* (Remacle et al., 2009). The *arg9* cells were grown in advance with supplementation of arginine, and thereafter were transferred to arginine-free medium for further growth for 24 h for repression of protein synthesis (Figure 3B, see –Arg, cf. *+Arg*, for *arg9*). This transfer simultaneously induced substantial accumulation of lipid droplets with an increase in the TG content from 6.6 to 24.7 mol%, relative to total lipids, on the basis of fatty acids (Figure 3C). Second, wild-type cells were cultured in the presence of CHI, which exhibited dose-dependent accumulation of lipid droplets (Figure 3D), i.e., 0.9 ± 0.7 and 3.7 ± 1.5 lipid droplets on a cell basis in untreated cells and cells treated with 10 μg·ml^−1^ CHI, respectively. Overall, the repression of two quite different metabolic processes for protein synthesis (arginine synthesis and the peptidyl transferase step in 70S ribosomes) was correlated with increased accumulated levels of TG and/or lipid droplets. These findings thus strongly supported the novel notion that repression of global protein synthesis is at least one of the key factors for the stimulation of accumulation of TG under S- or N-starved conditions.

We then investigated whether or not the *SAC1* and *SNRK2.2* genes are involved in the stimulation of the TG accumulation by S-starvation. Cells of the two disruptants as to the *SAC1* and *SNRK2.2* genes (*sac1* and *snrk2.2*, respectively), when mixotrophically grown in TAP medium under S-repleted conditions, contained 3 to 5 mol% TG relative to total lipids, on the basis of fatty acids, which was similar to the level in the case of the wild type (Figure 3E, see 0 h). Although S-starvation led to accumulation of lipid droplets at substantial levels in the disruptants of *sac1 and snrk2.2* (Figure 3F), characteristic mutational phenotypes as regards the TG content became evident on quantitative analysis of lipids (Figure 3E). The TG content on the basis of fatty acids was increased in the *sac1* disruptant to only 31.6 mol% relative to total lipids at 72 h, i.e., to a lower level than in WT (40.3 mol%, Figure 1C), with no further increase at 120 h. The effect of the *sac1* mutation of repression of TG accumulation could be demonstrated more definitely when the *sac1* disruptant was compared with the *SAC1*/*sac1* strain, which had accumulated TG to 53.2 mol% at 72 h, and thereafter maintained the increased content until at 120 h. Meanwhile, the TG content increased in the *snrk2.2* disruptant to 35.8 mol% at 72 h, and further to as high as 57.9 mol% at 120 h, i.e., to a higher level than in WT or the *SNRK2.2*/*snrk2.2* strain. Hence, the *snrk2.2* mutation, in contrast to the *sac1* one, had an impact of stimulation of TG accumulation.

### Effects of nutritional deficiency on the expression levels of the genes for TG synthesis in *C. reinhardtii*

Here, the possibility was investigated that expression of the genes that constitute the Kennedy pathway for the biosynthesis of TG is regulated under S-starved conditions (Figure 4), with the first focus on the *DGAT1* and *DGAT2* genes (Deng et al., 2012). Since the TG content increased steadily in wild-type cells of *C. reinhardtii* from the early to mid-phase of S-starvation (4–72 h, Figures 1B,C), the effects of S-starvation for 8 h on the transcript levels of the *DGAT1* and *DGAT2* genes were investigated by semi-quantitative RT-PCR (Figure 4). On S-starvation, *DGAT1* had little effect on the mRNA level. However, the *DGAT2* family (*DGTT1* to *4*), respectively, showed a higher mRNA level in S-starved cells than in control ones, the exception being *DGTT5* mRNA, which was below the detectable level irrespective of the ambient S-status (data not shown). The *DGAT2* family thus seemed to participate more positively than *DGAT1* in the increase of TG, at least during this short term S-starvation. Meanwhile, it was found that N-starved cells, similar to S-starved ones, exhibited an increased mRNA level of *DGTT1-4*, as a short-term response, with the level of *DGAT1* mRNA being hardly altered (Figure 4). In contrast, P-starved cells, as compared with control cells, exhibited little difference in the mRNA level of either *DGAT1* or *DGTT1-4* as a short-term response (Figure 4).

We then examined the transcript levels of the genes for the synthesis of phosphatidate (PA) from glycerol 3-phosphate (G3P) in the Kennedy pathway (Figure 4). This pathway starts with the first acylation of G3P at the *sn*−1 position, yielding lysophosphatidate (LPA), by G3P acyltransferase (GPAT), followed by the second acylation of LPA at the *sn*−2 position by lysophosphatidate acyltransferase (LPAAT), for construction of PA. It is predicted that *C. reinhardtii* possesses two genes that encode chloroplast- and ER-associated GPAT [designated here as *GPAT1* (a homolog of plastid *GPAT* of *A. thaliana*; Nishida et al., 1993) and *GPAT2* (a homolog of mammalian *GPAT3* or *A. thaliana GPAT9*; Nguyen et al., 2011), respectively]. Concerning the *LPAAT* gene, a homolog of plastid *LPAAT* of *A. thaliana* (Yu et al., 2004), but not of the ER-associated one, has been found. S-Starved cells showed higher mRNA levels for *GPAT1* and *LPAAT*, respectively, than control ones, with little effect on the level of *GPAT2* mRNA. N-Starved cells, distinct from S-starved ones, showed there was little impact on the mRNA level of either acyltransferase, while P-starved cells exhibited a higher level of only *GPAT1* mRNA compared to control cells.

Glycerol 3-phosphate (G3P), the first substrate in the Kennedy pathway (Figure 4), is generated by G3P dehydrogenase (GPDH) through reduction of dihydroxyacetone phosphate (DHAP). GPDH might thus be another key enzyme for induction of accumulation of TG. S-Starved cells, as compared with control ones, showed higher mRNA levels of CrGPDH2 and CrGPDH3, which are putative chloroplast isozymes (Herrera-Valencia et al., 2012). N-Starved cells, relative to the control ones, exhibited higher and lower levels of CrGPDH2 and CrGPDH3 mRNA, respectively, while P-starved cells showed remarkably lower mRNA levels of both chloroplast isozymes. On the other hand, mRNA of CrGPDH1, a probable cytoplasmic isozyme (Herrera-Valencia et al., 2012), was undetectable in our study, irrespective of the growth conditions (data not shown). Overall, it was found that the expression levels of the genes for TG synthesis in Figure 4, with the exceptions of *DGAT1* and *GPAT2*, are up-regulated under S-starved conditions, but that the genes subjected to up-regulation are limited to the *DGTT1-4* and *GPDH2* ones under N-starved conditions (Table 1).

**Table 1:**
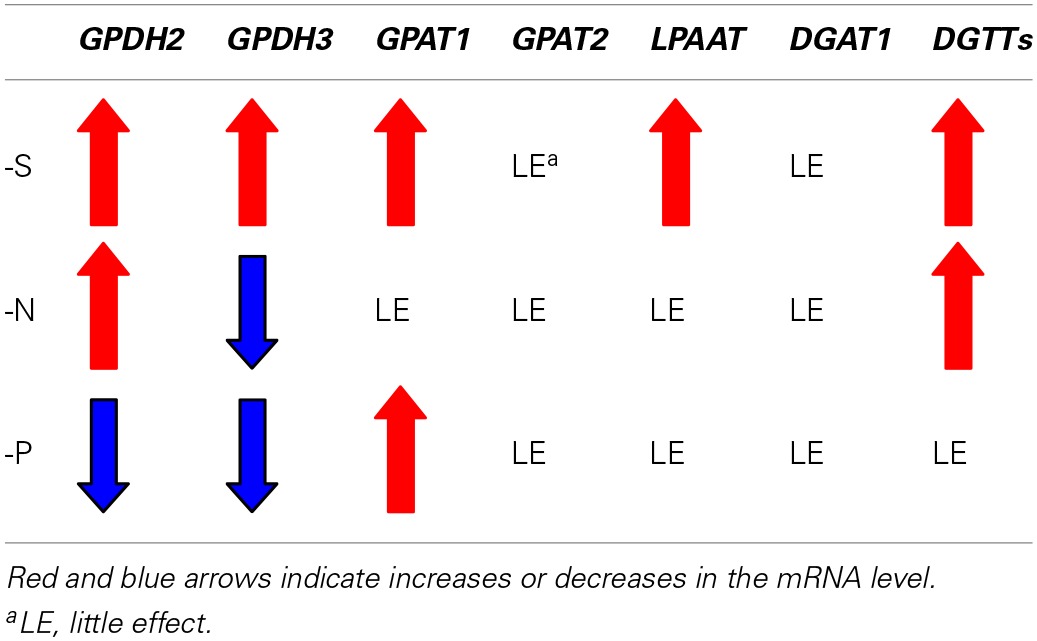
**Summary of effects of S-, N-, or P-starvation on the levels of transcripts for the genes for TG synthesis**.

*Red and blue arrows indicate increases or decreases in the mRNA level*.

*^a^LE, little effect*.

**Figure 5:**
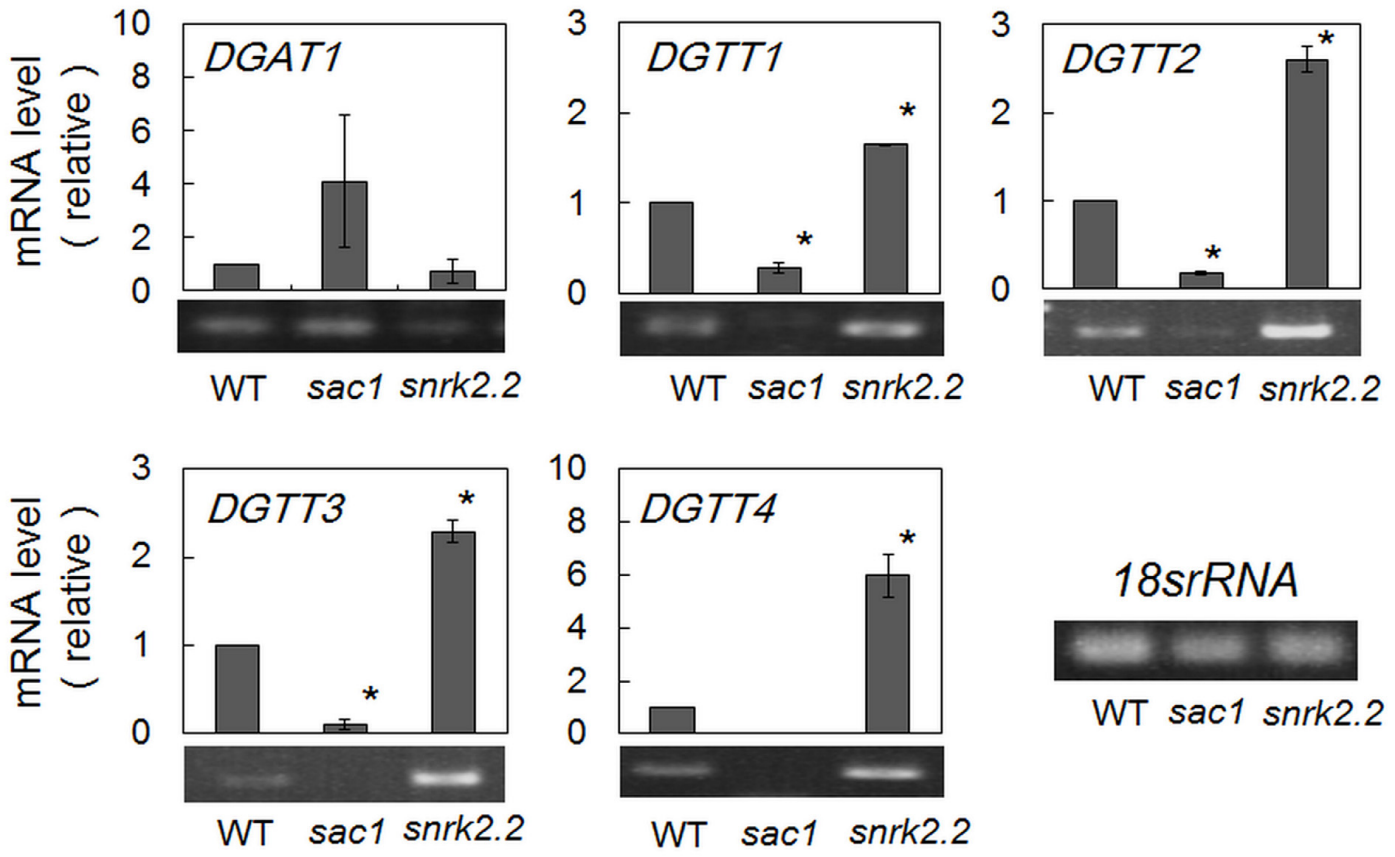
**Semi-quantitative RT-PCR analysis of mRNA levels of the DGAT1-4 genes in WT, and *sac1* and *snrk2.2* cells after S-starvation for 8 h**. The values were estimated, as described in the legend to Figure 5, and are the averages ± SE for three distinct groups of data. The significance of differences was evaluated by means of Student's *t*-test. ^*^P < 0.05.

The wild-type cells, when starved of S, showed induction of expression of the genes for DGTT1-4 at particularly high levels. The expression levels of *DGTT1-4* under S-starved conditions were then examined in *sac1* and *snrk2.2* disruptants (Figure 5). The *sac1* disruptant, which was repressed as to TG accumulation, was defective in the up-regulation of the mRNA levels of *DGTT1-4*. In contrast, the *snrk2.2* disruptant, which was stimulated as to TG accumulation, showed much stronger up-regulation of the levels of the *DGTT1-4* mRNAs. On the other hand, the *snrk2.2* mutation had little impact on the mRNA level of *DGAT1*. However, the *sac1* mutation brought about an increased level of *DGAT1* mRNA, implying some compensatory mechanism for the failure in the induction system as to expression of the *DGTT1-4* genes.

## Discussion

### Establishment of S-starvation as a stress condition that induces TG accumulation in green algae

S-Starvation, distinct from N-starvation, has hardly attracted researchers' attention as a stress condition for algal TG production. *Chlorella ellipsoidea* cells showed an increase in the content of total lipids during S-starvation, however, the responsible lipid class(es) were not specified (Otsuka, 1961). The TG content was increased in *C. reinhardtii* cells under combinational stress conditions of S-starvation and anaerobicity, which obscured the causal environmental factor for the TG accumulation (Matthew et al., 2009). During the course of our study, two reports regarding the effects of S-starvation on the accumulated level of TG in *C. reinhardtii* were published, however, the results were incompatible: Fan et al. (2012) found little stimulation of TG accumulation after 3-day starvation of S, whereas Boyle et al. (2012) reported induction of TG accumulation after 2-day starvation. Cakmak et al. (2012) reported that S-starved cells of *C. reinhardtii* accumulated TG, and that, in contrast to our results, its attained level was higher than in N-starved cells. In the last study, TG was quantified with the use of a spectrum band by Fourier transform infrared spectroscopy (FTIR), which was attributed to ester group vibration of TG. However, they analyzed the dried cells containing the other cellular lipids, ester group vibration of which would interfere with that of TG. In this context, our detailed time-scale study, although consistent with the work by Boyle et al. (2012), is the first that demonstrated conclusively that S-starvation causes TG to steadily accumulate in *C. reinhartdii*, and in another green alga, *C. kessleri*, as well (Figures 1B–E). Moreover, our study is novel in definitely demonstrating that stimulation of TG accumulation is a specific response to S- or N-starvation, but not a general response to nutritional deficiency (Figure 1C).

### Metabolic alteration for stimulation of TG synthesis in *C. reinhardtii* cells during S-starvation

Prevention of TG accumulation by cerulenin (Figure 3A) would suggest that acyl-groups for TG synthesis are supplied predominantly through de novo synthesis of fatty acids in S-starved cells, as was previously reported for N-starved cells (Fan et al., 2011). In line with this idea, the content of glycerate 3-phosphate, a precursor of acetyl-CoA that provides building blocks for fatty acid synthetase, was 3.7-fold elevated in S-starved cells of *C. reinhardtii* (Bölling and Fiehn, 2005). Simultaneously, the cellular content of G3P increased by 3.3-fold with a decrease in the DHAP content to 0.6-fold (Bölling and Fiehn, 2005). S-Starvation thus seemed to stimulate G3P synthesis through enhancement of the transcript levels of CrGPDH2 and 3 (Figure 4), and, accordingly, the activity of GPDH. S-Starved cells must perform photosynthesis to accumulate TG (Figure 3A), which would reflect requirement of photosynthesis for production of chemical energy and fixed carbon for synthesis of acyl-groups and G3P.

As reflected in the delayed growth of S-starved cells (Figure 1A), *C. reinhardtii* cells starved of S are known to exhibit down-regulated photosynthesis (Wykoff et al., 1998), which would help repress the production of reactive oxygen species (ROS; González-Ballester et al., 2010). Simultaneously, activated synthesis of acyl-groups and G3P would also contribute to repressed production of ROS by facilitating the consumption of excessive reducing power and the resultant prevention of over-reduction of cells in a redox state (Hu et al., 2008; Li et al., 2012). It is likely that starch accumulation (Figure 1F) also contributes to repression of ROS production. Such regulation of the energy balance would meet the algal requirements for TG and starch, as intracellular carbon- and energy-sources, just when algal cells resume growth upon relief from S-starvation. The metabolic regulation for allocating carbon between TG and starch syntheses is a future subject.

Meanwhile, *in C. reinhardtii* cells under normal conditions, the cellular content of total protein relative to dry cell weight reached as high as 48% (w/w), cf., lipids and carbohydrates that only represent 21 and 17%, respectively (Becker, 2007). TG accumulation stimulated by the *arg9* mutation or application of CHI (Figures 3C,D) could reflect diversion of a predominant metabolic carbon-flux from protein synthesis to the synthesis of carbon-storage compounds including TG. Repression of global protein synthesis might thus be one of the key factors in the stimulation of TG synthesis under S- or N-starved conditions. These observations in turn indicated that pre-existing enzymes for TG synthesis, by themselves, could contribute to TG synthesis. Compatible with this idea, the *sac1* disruptant that was unable to up-regulate the expression levels of *DGTT1-4* could still elevate the TG content (Figures 3E, 5). In this context, it seemed that the complete repression of TG accumulation in S-starved cells of *C. reinhardtii* on the application of CHI (Figure 3A) was caused by some secondary effect of CHI, which would become apparent only under severe S-starved conditions, i.e., not under normal nutritional ones. Similar interpretation might be necessary, concerning inhibition of TG accumulation in N-starved *C. reinhardtii* cells (Fan et al., 2011). Again, it should be emphasized that repression of protein synthesis could trigger TG accumulation in S-or N-starved cells of *C. reinhardtii*. Induction of TG accumulation by S-starvation would be conserved in a wide range of algal species in terms of their substantial protein contents, i.e., equal to or more than half of the dry cell weight (Becker, 2007).

### Regulation of expression levels of the genes responsible for the elevation of TG synthesis in *C. reinhardtii* cells under S-starved conditions

Expression patterns of genes for TG synthesis have been extensively examined in N-starved *C. reinhardtii* cells (see below), but infrequently in S-starved ones. Positive participation of the *DGAT2* family, but not of *DGAT1*, was suggested in TG accumulation during short term S-starvation (Figure 5 and Table 1). Long-term S-starvation for 2 days induced up-regulation of the respective mRNA levels of *DGTT1* and *DGAT1* in *C. reinhardtii*, although the effect was much less remarkable on that of *DGAT1* (Boyle et al., 2012). It can thus be proposed that the induction of *DGTT1* starts at an early stage of starvation (at 8 h at latest), and that a time-dependent alteration in the level of *DGAT1* mRNA should be investigated in more detail to understand regulation of the expression of *DGAT1*.

The elevation of the transcript levels of the genes for chloroplast-type GPDH, GPAT, and LPAAT, and ER-type DGTT1-4 in S-starved cells (Figure 4 and Table 1) implied enhancement of the corresponding enzymatic activities for facilitation of PA synthesis in chloroplasts and subsequent TG synthesis at the ER. Intriguingly, C16 acids exclusively occupied the *sn*−2 position of TG in N-starved cells of *C. reinhardtii*, which inferred predominant synthesis of a DG moiety for TG synthesis in chloroplasts (Fan et al., 2011). The similar fatty acid profile of TG for S- starved cells to that for N-starved ones (Figure 2) would also imply synthesis of a DG moiety for TG synthesis mainly at chloroplasts in S-starved cells.

The *sac1* and *snrk2.2* mutations led to repression and facilitation, respectively, in up-regulation of the *DGTT1-4* genes under S-starved conditions (Figure 5). The arylsulfatase gene (*ARS1*) previously showed regulatory properties similar to those of the *DGAT1-4* genes: the *ARS1* mRNA level is elevated in cells of *C. reinhardtii* during S-starvation, and the elevated level becomes lower and higher with the *sac1* and *snrk2.2* mutations, respectively (Davies et al., 1996; Ravina et al., 2002). It is likely that the SAC1 protein acts as a positive regulator under S-starved conditions, causing stimulated expression of the *DGTT1 to 4* genes as well as the *ARS1* one. The pronouncedly high accumulation of TG in *sac1*/*SAC1* (Figure 3E) might be caused by some mechanism that elevates the expression level of *SAC1*, which has yet to be investigated. Contrarily, it might be that the SNRK2.2 protein acts as a negative regulator for repression of expression of the *DGTT1-4* genes, as well as of that of the *ARS1* one. It should be emphasized that these actions of *SAC1* and *SNRK2.2* could be directly related to the accumulation level of TG in S-starved cells (Figure 3E). Collectively, it was newly found that, in S-starved cells, a series of genes for chloroplast PA synthesis, similar to those for DGTTs, are up-regulated in expression levels, and that *SAC1* and *SNRK2.2* are greatly responsible for the up-regulated levels of *DGTT1-4* expression, and consequently of TG.

Numerous groups have attempted to enhance the production level of algal TG to one suitable for industrial application, through manipulation of the genes related to TG synthesis, including those of DGAT and acetyl-CoA carboxylase, which catalyze the rate-limiting step of fatty acid synthesis (e.g., Deng et al., 2012). However, this kind of strategy has limitations in the level of stimulation of TG synthesis through overexpresssion of individual genes. Moreover, the notion of the establishment of a predominant metabolic carbon-flow to TG synthesis has been overlooked. In this context, this study is novel in the discovery that regulatory genes, *SAC1* and *SNRK2.2*, can be used to simultaneously manipulate the expression levels of a series of *DGAT2* genes, and that this aberrant regulation takes place under S-starved conditions, where a prominently large carbon flux into protein synthesis could be deviated to TG synthesis.

### Different aspects of TG accumulation under N-or P-starved conditions from those under S-starved conditions

It seems likely that N-starved cells as well as S-starved ones are repressed in protein synthesis for stimulation of TG synthesis (Figure 3B). Moreover, up-regulation as to mRNA levels of DGTT1-4 and GPDH2 was observed also in N-starved cells (Figure 4 and Table 1), as was previously reported (Miller et al., 2010; Boyle et al., 2012; Msanne et al., 2012; Blaby et al., 2013; Ramanan et al., 2013). However, N-starved cells of *C. reinhardtii*, distinct from S-starved ones, showed no increases in the mRNA levels of *GPDH3, GPAT1*, and *LPAAT* (Figure 4 and Table 1). Blaby et al. (2013) also reported no significant increases for *GPDH3* and *GPAT1*. Thus, we could interpret that N-starved cells, despite their even higher TG content than that of S-starved ones, exhibited no positive evidence of reinforcement of these three enzyme functions in PA synthesis through confirmation of previous reports (*GPDH3, GPAT1*) and with a new information (*LPAAT*). These results might reflect the possible greater availability of PA for TG synthesis in N-starved cells than in S-starved ones. Photosynthetic organisms show decreased synthesis of phospholipids and SQDG under P- and S-starved conditions, respectively, because of a shortage of nutrients for their synthesis (Sato, 2004). N-Containing lipids such as phosphatidylethanolamine and diacylglyceryltrimethylhomoserine totally occupy as much as 30 mol% of total lipids in cells of *C. reinhardtii* (Sugimoto et al., 2008). Repression of the synthesis of N-containing lipids at ER would support preferential metabolic flow of G3P and acyl-groups into PA synthesis at chloroplasts in N-starved cells. This scenario, however, needs experimental verification.

P-Starvation for a short term had little effect on the mRNAs of *DGTT1-4* (Figure 4 and Table 1). It was previously found that, even after long-term P-starvation for 2 days, the *DGTT1* mRNA level hardly changed (Boyle et al., 2012). It thus seemed likely that P-starvation has little impact on *DGAT2* expression throughout a P-starvation period, in line with no stimulated accumulation of TG (Figure 1C). Meanwhile, P-starvation caused the mRNA levels of *GPAT1*, and *GPDH 2* and *3* to increase and decrease, respectively (Figure 4 and Table 1). The decreased levels of *GPDH2* and *3* mRNAs would reflect the low requirement of P-starved cells for G3P, owing to repressed phospholipid synthesis. The increased level of *GPAT1* mRNA would lead to enhancement of GPAT activity, thereby drawing G3P from its cellular pool preferentially into the pathway of polar lipid synthesis for cell proliferation. We can thus suggest deficient-nutrient specific regulatory mechanisms, as to the expression levels of the genes for TG synthesis.

Different regulation of gene expression for TG synthesis between S- and N-starved *C. reinhardtii* cells was previously reported for phospholipid:diacylglycerol acyltransferase (PDAT). The level of PDAT mRNA was little affected by S-starvation, but increased during N-starvation. Consistently, disruption of the *PDAT* gene had no impact on TG accumulation in S-starved cells, whereas it caused a 25% decrease in the accumulated level of TG in N-starved cells (Boyle et al., 2012).

Overall, our study demonstrated two novel key points that contribute to stimulation of TG synthesis under S-starved conditions. The first is repression of protein synthesis by S-starvation, probably to make the metabolic pool larger, as to fixed carbon and chemical energy for the synthesis of other organic compounds including TG. The second is induction of expression of a series of genes for TG synthesis at properly high levels through the actions of the *SAC1* and *SNRK2.2* genes, which thereby draws metabolic carbon flow preferentially into TG synthesis. This study has provided a basic architecture for a comprehensive understanding of the regulatory mechanism, from both metabolic and molecular aspects, by which TG synthesis is stimulated by S-starvation, and would further contribute to elucidation of the regulatory mechanisms under other stress conditions such as N-starvation. Future study along these lines will allow us to develop a strategy for the production of algal TG on a large scale that is practical for industrial application.

### Conflict of interest statement

The authors declare that the research was conducted in the absence of any commercial or financial relationships that could be construed as a potential conflict of interest.

## References

[B1] Athenstaedt K. Daum G. (2006). The life cycle of neutral lipids: synthesis, storage and degradation. Cell. Mol. Life Sci. 63, 1355–1369 10.1007/s00018-006-6016-8 16649142 PMC11136409

[B2] Becker E. W. (2007). Micro-algae as a source of protein. Biotechnol. Adv. 25, 207–210 10.1016/j.biotechadv.2006.11.002 17196357

[B3] Blaby I. K. Glaesener A. G. Mettler T. Fitz-Gibbon S. T. Gallaher S. D. Liu B. (2013). Systems-level analysis of nitrogen starvation-induced modifications of carbon metabolism in a *Chlamydomonas reinhardtii* starchless mutant. Plant Cell 25, 4305–4323 10.1105/tpc.113.117580 24280389 PMC3875720

[B4] Bligh E. G. Dyer W. J. (1959). A rapid method of total lipid extraction and purification. Can. J. Biochem. Physiol. 37, 911–917 10.1139/o59-099 13671378

[B5] Bölling C. Fiehn O. (2005). Metabolite profiling of *Chlamydomonas reinhardtii* under nutrient deprivation. Plant Physiol. 139, 1995–2005 10.1104/pp.105.071589 16306140 PMC1310576

[B6] Boyle N. R. Page M. D. Liu B. Blaby I. K. Casero D. Kropat J. (2012). Three acyltransferases and nitrogen-responsive regulator are implicated in nitrogen starvation-induced triacylglycerol accumulation in *Chlamydomonas*. J. Biol. Chem. 287, 15811–15825 10.1074/jbc.M111.334052 22403401 PMC3346115

[B7] Cakmak T. Angun P. Demiray Y. E. Ozkan A. D. Elibol Z. Tekinay T. (2012). Differential effects of nitrogen and sulfur deprivation on growth and biodiesel feedstock production of *Chlamydomonas reinhardtii*. Biotechnol. Bioeng. 109, 1947–1957 10.1002/bit.24474 22383222

[B8] Davies J. P. Yildiz F. H. Grossman A. (1996). *Sacl*, a putative regulator that is critical for survival of *Chlamydomonas reinhardtii* during sulfur deprivation. EMBO J. 15, 2150–2159 8641280 PMC450137

[B9] Davies J. P. Yildiz F. H. Grossman A. R. (1999). *Sac3*, an *Snf1*-like serine/threonine kinase that positively and negatively regulates the responses of *Chlamydomonas* to sulfur limitation. Plant Cell 11, 1179–1190 10368187 10.1105/tpc.11.6.1179 PMC144238

[B10] Deng X.-D. Gu B. Li Y.-J. Hu X.-W. Guo J.-C. Fei X.-W. (2012). The roles of acyl-CoA: diacylglycerol acyltransferase 2 genes in the biosynthesis of triacylglycerols by the green algae *Chlamydomonas reinhardtii*. Mol. Plant 5, 945–947 10.1093/mp/sss040 22511604

[B11] Dunstan G. A. Volkman J. K. Barrett S. M. Garland C. D. (1993). Changes in the lipid composition and maximisation of the polyunsaturated fatty acid content of three microalgae grown in mass culture. J. Appl. Phycol. 5, 71–83

[B12] Durrett T. P. Benning C. Ohlrogge J. (2008). Plant triacylglycerols as feedstocks for the production of biofuels. Plant J. 54, 593–607 10.1111/j.1365-313X.2008.03492.x 18476866

[B13] Fan J. Andre C. Xu C. (2011). A chloroplast pathway for the de novo biosynthesis of triacylglycerol in *Chlamydomonas reinhardtii*. FEBS Lett. 585, 1985–1991 10.1016/j.febslet.2011.05.018 21575636

[B14] Fan J. Yan C. Andre C. Shanklin J. Schwender J. Xu C. (2012). Oil accumulation is controlled by carbon precursor supply for fatty acid synthesis in *Chlamydomonas reinhardtii*. Plant Cell Physiol. 53, 1380–1390 10.1093/pcp/pcs082 22642988

[B15] González-Ballester D. Casero D. Cokus S. Pellegrini M. Merchant S. S. Grossman A. R. (2010). RNA-Seq analysis of sulfur-deprived *Chlamydomonas* cells reveals aspects of acclimation critical for cell survival. Plant Cell 22, 2058–2084 10.1105/tpc.109.071167 20587772 PMC2910963

[B16] Gorman D. S. Levine R. P. (1965). Cytochrome f and plastocyanin: their sequence in the photosynthetic electron transport chain of *Chlamydomonas reinhardi*. Proc. Natl. Acad. Sci. U.S.A. 54, 1665–1669 4379719 10.1073/pnas.54.6.1665 PMC300531

[B17] Grimme L. H. Boardman N. K. (1972). Photochemical activities of a particle fraction P_1_ obtained from the green alga *Chlorella fusca*. Biochem. Biophys. Res. Commun. 49, 1617–1623 4404797 10.1016/0006-291x(72)90527-x

[B18] Herrera-Valencia V. A. Macario-González L. A. Casais-Molina M. L. Beltran-Aguilar A. G. Peraza-Echeverría S. (2012). In silico cloning and characterization of the glycerol-3-phosphate dehydrogenase (GPDH) gene family in the green microalga *Chlamydomonas reinhardtii*. Curr. Microbiol. 64, 477–485 10.1007/s00284-012-0095-6 22358185

[B19] Hu Q. Sommerfeld M. Jarvis E. Ghirardi M. Posewitz M. Seibert M. (2008). Microalgal triacylglycerols as feedstocks for biofuel production: perspectives and advances. Plant J. 54, 621–639 10.1111/j.1365-313X.2008.03492.x 18476868

[B20] Hubberten H. M. Drozd A. Tran B. V. Hesse H. Hoefgen R. (2012). Local and systemic regulation of sulfur homeostasis in roots of *Arabidopsis thaliana*. Plant J. 72, 625–635 10.1111/j.1365-313X.2012.05105.x 22775482

[B21] Izumo A. Fujiwara S. Sakurai T. Ball S. G. Ishii Y. Ono H. (2011). Effects of granule-bound starch synthase 1-defective mutant on the morphology and structure of pyrenoidal starch in *Chlamydomonas*. Plant Sci. 180, 238–245 10.1016/j.plantsci.2010.08.014 21421366

[B22] Khotimchenko S. V. Yakovleva I. M. (2004). Effect of solar irradiance on lipids of the green alga *Ulva fenestrata Postels et Ruprecht*. Bot. Mar. 47, 395–401 10.1515/BOT.2004.050

[B23] Kohlwein S. D. Henry S. A. (2011). Coordination of storage lipid synthesis and membrane biogenesis: evidence for cross-talk between triacylglycerol metabolism and phosphatidylinositol synthesis. J. Biol. Chem. 286, 1696–1708 10.1074/jbc.M110.172296 20972264 PMC3023464

[B24] Li X. Moellering E. R. Liu B. Johnny C. Fedewa M. Sears B. B. (2012). A galactoglycerolipid lipase is required for triacylglycerol accumulation and survival following nitrogen deprivation in *Chlamydomonas reinhardtii*. Plant Cell 24, 4670–4686 10.1105/tpc.112.105106 23161887 PMC3531859

[B25] Los D. A. Ray M. K. Murata N. (1997). Differences in the control of the temperature-dependent expression of four genes for desaturases in *Synechocystis* sp. PCC 6803. Mol. Microbiol. 25, 1167–1175 10.1046/j.1365-2958.1997.5641912.x 9350872

[B26] Matthew T. Zhou W. Rupprecht J. Lim L. Thomas-Hall S. R. Doebbe A. (2009). The metabolome of *Chlamydomonas reinhardtii* following induction of anaerobic H_2_ production by sulfur depletion. J. Biol. Chem. 284, 23415–23425 10.1074/jbc.M109.003541 19478077 PMC2749115

[B27] Merchant S. S. Kropat J. Liu B. Shaw J. Warakanont J. (2012). TAG, You're it! Chlamydomonas as a reference organism for understanding algal triacylglycerol accumulation. Curr. Opin. Biotechnol. 23, 352–363 10.1016/j.copbio.2011.12.001 22209109

[B28] Merchant S. S. Prochnik S. E. Vallon O. Harris E. H. Karpowicz S. J. Witman G. B. (2007). The *Chlamydomonas* genome reveals the evolution of key animal and plant functions. Science 318, 245–250 10.1126/science.1143609 17932292 PMC2875087

[B29] Miller R. Wu G. Deshpande R. R. Vieler A. Gärtner K. Li X. (2010). Changes in transcript abundance in *Chlamydomonas reinhardtii* following nitrogen deprivation predict diversion of metabolism. Plant Physiol. 154, 1737–1752 10.1104/pp.110.165159 20935180 PMC2996024

[B30] Minoda A. Sonoike K. Okada K. Sato N. Tsuzuki M. (2003). Decrease in the efficiency of the electron donation to tyrosine Z of photosystem II in an SQDG-deficient mutant of *Chlamydomonas*. FEBS Lett. 553, 109–112 10.1016/S0014-5793(03)00981-5 14550556

[B31] Moseley J. L. Gonzalez-Ballester D. Pootakham W. Bailey S. Grossman A. R. (2009). Genetic interactions between regulators of *Chlamydomonas* phosphorus and sulfur deprivation responses. Genetics 181, 889–905 10.1534/genetics.108.099382 19087952 PMC2651062

[B32] Msanne J. Xu D. Konda A. R. Casas-Mollano J. A. Awada T. Cahoon E. B. (2012). Metabolic and gene expression changes triggered by nitrogen deprivation in the photoautotrophically grown microalgae *Chlamydomonas reinhardtii* and *Coccomyxa* sp. C-169. Phytochemistry 75, 50–59 10.1016/j.phytochem.2011.12.007 22226037

[B33] Nguyen H. M. Baudet M. Cuine S. Adriano J.-M. Barthe D. Billon E. (2011). Proteomic profiling of oil bodies isolated from the unicellular green microalga *Chlamydomonas reinhardtii*: with focus on proteins involved in lipid metabolism. Proteomics 11, 4266–4273 10.1002/pmic.201100114 21928291

[B34] Nikiforova V. Freitag J. Kempa S. Adamik M. Hesse H. Hoefgen R. (2003). Transcriptome analysis of sulfur depletion in *Arabidopsis thaliana*: interlacing of biosynthetic pathways provides response specificity. Plant J. 33, 633–650 10.1046/j.1365-313X.2003.01657.x 12609038

[B35] Nishida I. Tasaka Y. Shiraishi H. Murata N. (1993). The gene and the RNA for the precursor to the plastid-located glycerol-3-phosphate acyltransferase of *Arabidopsis thaliana*. Plant Mol. Biol. 21, 267–277 10.1007/BF00019943 7678766

[B36] Otsuka H. (1961). Changes of lipid and carbohydrate contents of *Chlorella* cells during the sulfur starvation, as studied by the technique of synchronous culture. J. Gen. Appl. Microbiol. 7, 72–77 10.2323/jgam.7.72

[B37] Pootakham W. Gonzalez-Ballester D. Grossman A. R. (2010). Identification and regulation of plasma membrane sulfate transporters in *Chlamydomonas*. Plant Physiol. 153, 1653–1668 10.1104/pp.110.157875 20498339 PMC2923900

[B38] Quettier A. L. Eastmond P. J. (2009). Storage oil hydrolysis during early seedling growth. Plant Physiol. Biochem. 47, 485–490 10.1016/j.plaphy.2008.12.005 19136267

[B39] Rajakumari S. Rajasekharan R. Daum G. (2010). Triacylglycerol lipolysis is linked to sphingolipid and phospholipid metabolism of the yeast, *Saccharomyces cerevisiae*. Biochim. Biophys. Acta 1801, 1314–1322 10.1016/j.bbalip.2010.08.004 20727985

[B40] Ramanan R. Kim B. H. Cho D. H. Ko S. R. Oh H. M. Kim H. S. (2013). Lipid droplet synthesis is limited by acetate availability in starchless mutant of *Chlamydomonas reinhardtii*. FEBS Lett. 587, 370–377 10.1016/j.febslet.2012.12.020 23313852

[B41] Ravina C. G. Chang C.-I. Tsakraklides G. P. McDermott J. P. Vega J. M. Leustek T. (2002). The *sac* mutants of *Chlamydomonas reinhardtii* reveal transcriptional and posttranscriptional control of cysteine biosynthesis. Plant Physiol. 130, 2076–2084 10.1104/pp.012484 12481091 PMC166719

[B42] Remacle C. Cline S. Boutaffala L. Gabilly S. Larosa V. Barbieri M. R. (2009). The *ARG9* gene encodes the plastid-resident N-acetyl ornithine aminotransferase in the green alga *Chlamydomonas reinhardtii*. Eukaryot. Cell 8, 1460–1463 10.1128/EC.00108-09 19617392 PMC2747819

[B43] Rochaix J. D. (1995). *Chlamydomonas reinhardtii* as the photosynthetic yeast. Annu. Rev. Genet. 29, 209–230 10.1146/annurev.ge.29.120195.001233 8825474

[B44] Sato N. (2004). Roles of the acidic lipids sulfoquinovosyl diacylglycerol and phosphatidylglycerol in photosynthesis: their specificity and evolution. J. Plant Res. 117, 495–505 10.1007/s10265-004-0183-1 15538651

[B45] Sato N. Aoki M. Maru Y. Sonoike K. Minoda A. Tsuzuki M. (2003a). Involvement of sulfoquinovosyl diacylglycerol in the structural integrity and heat-tolerance of photosystem II. Planta 217, 245–251 10.1007/s00425-003-0992-9 12783332

[B46] Sato N. Tsuzuki M. Kawaguchi A. (2003b). Glycerolipid synthesis in *Chlorella kessleri* 11h. I. Existence of a eukaryotic pathway. Biochim. Biophys. Acta 1633, 27–34 10.1016/S1388-1981(03)00069-6 12842192

[B47] Sato N. Tsuzuki M. Matsuda Y. Ehara T. Osafune T. Kawaguchi A. (1995). Isolation and characterization of mutants affected in lipid metabolism of *Chlamydomonas reinhardtii*. Eur. J. Biochem. 230, 987–993 10.1111/j.1432-1033.1995.0987g.x 7601163

[B48] Siaut M. Cuiné S. Cagnon C. Fessler B. Nguyen M. Carrier P. (2011). Oil accumulation in the model green alga *Chlamydomonas reinhardtii*: characterization, variability between common laboratory strains and relationship with starch reserves. BMC Biotechnol. 11:7 10.1186/1472-6750-11-7 21255402 PMC3036615

[B49] Sueoka N. (1960). Mitotic replication of deoxyribonucleic acid in *Chlamydomonas reinhardti*. Proc. Natl. Acad. Sci. U.S.A. 46, 83–89 16590601 10.1073/pnas.46.1.83 PMC285018

[B50] Sugimoto K. Midorikawa T. Tsuzuki M. Sato N. (2008). Upregulation of PG synthesis on sulfur-starvation for PSI in *Chlamydomonas*. Biochem. Biophys. Res. Commun. 369, 660–665 10.1016/j.bbrc.2008.02.058 18295600

[B51] Sugimoto K. Sato N. Tsuzuki M. (2007). Utilization of a chloroplast membrane sulfolipid as a major internal sulfur source for protein synthesis in the early phase of sulfur starvation in *Chlamydomonas reinhardtii*. FEBS Lett. 581, 4519–4522 10.1016/j.febslet.2007.08.035 17765894

[B52] Sugimoto K. Tsuzuki M. Sato N. (2010). Regulation of synthesis and degradation of a sulfolipid under sulfur-starved conditions and its physiological significance in *Chlamydomonas reinhardtii*. New Phytol. 185, 676–686 10.1111/j.1469-8137.2009.03115.x 20003074

[B53] Tabei Y. Okada K. Tsuzuki M. (2007). Sll1330 controls the expression of glycolytic genes in *Synechocystis* sp. PCC 6803. Biochem. Biophys. Res. Commun. 355, 1045–1050 10.1016/j.bbrc.2007.02.065 17331473

[B54] Teramoto H. Nakamori A. Minagawa J. Ono T. (2002). Light-intensity-dependent expression of Lhc gene family encoding light-harvesting chlorophyll-a/b proteins of photosystem II in *Chlamydomonas reinhardtii*. Plant Physiol. 130, 325–333 10.1104/pp.004622 12226512 PMC166565

[B55] Toepel J. Albaum S. P. Arvidsson S. Goesmann A. la Russa M. Rogge K. (2011). Construction and evaluation of a whole genome microarray of *Chlamydomonas reinhardtii*. BMC Genomics 25:579 10.1186/1471-2164-12-579 22118351 PMC3235179

[B56] Wykoff D. D. Davies J. P. Melis A. Grossman A. R. (1998). The regulation of photosynthetic electron transport during nutrient deprivation in *Chlamydomonas reinhardtii*. Plant Physiol. 117, 129–139 10.1104/pp.117.1.129 9576782 PMC34996

[B57] Yu B. Wakao S. Fan J. Benning C. (2004). Loss of plastidic lysophosphatidic acid acyltransferase causes embryo-lethality in *Arabidopsis*. Plant Cell Physiol. 45, 503–510 10.1093/pcp/pch064 15169931

[B58] Zhang Z. Shrager J. Jain M. Chang C.-W. Vallon O. Grossman A. R. (2004). Insights into the survival of *Chlamydomonas reinhardtii* during sulfur starvation based on microarray analysis of gene expression. Eukaryot. Cell 3, 1331–1348 10.1128/EC.3.5.1331-1348.2004 15470261 PMC522608

